# The crosstalk role of CDKN2A between tumor progression and cuproptosis resistance in colorectal cancer

**DOI:** 10.18632/aging.205945

**Published:** 2024-06-17

**Authors:** Xifu Cheng, Famin Yang, Yuanheng Li, Yuke Cao, Meng Zhang, Jiameng JI, Yuxiao Bai, Qing Li, Qiongfang Yu, Dian Gao

**Affiliations:** 1 Department of Gastroenterology and Hepatology, The Second Affiliated Hospital, Jiangxi Medical College, Nanchang University, Nanchang 330006, China; 2 Department of Pathogen Biology and Immunology, School of Basic Medical Sciences, Jiangxi Medical College, Nanchang University, Nanchang 330006, China; 3 School of Ophthalmology and Optometry, Jiangxi Medical College, Nanchang University, Nanchang 330006, China; 4 Queen Mary School, Jiangxi Medical College, Nanchang University, Nanchang 330031, China; 5 Department of Gastroenterology and Hepatology, Shenzhen Hospital of Southern Medical University, Shenzhen 518000, China; 6 Department of Oncology, The Second Affiliated Hospital, Jiangxi Medical College, Nanchang University, Nanchang 330006, China

**Keywords:** cuproptosis, CDKN2A, bioinformatics, metabolism, radiation therapy

## Abstract

Background: Cuproptosis is a type of cell death characterized by excessive copper-lipid reactions in the tricarboxylic acid cycle, resulting in protein toxicity stress and cell death. Although known as a cuproptosis inhibitor through CRISPR-Cas9 screening, the role of cyclin-dependent kinase inhibitor 2A (CDKN2A) in cuproptosis resistance and its connection to tumor development remains unclear.

Methods: In this study, we combined single-cell sequencing, spatial transcriptomics, pathological image analysis, TCGA multi-omics analysis and *in vitro* experimental validation to comprehensively investigate CDKN2A distribution, expression, epigenetic modification, regulation and genomic features in colorectal cancer cells. We further explored the associations between CDKN2A and cellular pathway, immune infiltration and spatial signal communication.

Results: Our findings showed an increasing trend in cuproptosis in the trajectory of tumor progression, accompanied by an upward trend of CDKN2A. CDKN2A underwent transcriptional activation by MEF2D and via the SNHG7/miR-133b axis, upregulating glycolysis, copper metabolism and copper ion efflux. CDKN2A likely drives epithelial-mesenchymal transition (EMT) and progression by activating Wnt signaling. CDKN2A is associated with high genomic instability and sensitivity to radiation and chemotherapy. Tumor regions expressing CDKN2A exhibit distinctive SPP1+ tumor-associated macrophage (TAM) infiltration and MMP7 enrichment, along with unique signaling crosstalk with adjacent areas.

Conclusions: CDKN2A mediates cuproptosis resistance through regulating glycolysis and copper homeostasis, accompanied by a malignant phenotype and pro-tumor niche. Radiation and chemotherapy are expected to potentially serve as therapeutic approaches for cuproptosis-resistant colorectal cancer with high CDKN2A expression.

## INTRODUCTION

Colorectal cancer is a prevalent gastrointestinal malignancy with increasing incidence worldwide. It ranks third in cancer diagnoses and second in cancer mortality, posing a significant health burden [1]. Most patients are diagnosed at late stages due to initial subtle symptoms, resulting in poor prognosis [2]. Elucidating the mechanisms underlying colorectal cancer progression is thus vital. Recent omics studies have provided insights into regulatory pathways involved in colorectal tumorigenesis and progression [3].

Copper plays critical roles in biological processes, including iron metabolism, mitochondrial respiration, antioxidant response, and angiogenesis. Both copper excess and deficiency can be detrimental [4]. In cancers, elevated copper promotes proliferation, invasion, metastasis, and angiogenesis [3]. Excess copper can cause protein toxicity stress and trigger a distinct cell death pathway called cuproptosis [5]. A number of studies have investigated the role of some cuproptosis-related genes in tumors [6]. The dysregulation of CDKN2A promotes resistance to cuproptosis, but the mechanisms are still unclear, with limited related research [7, 8]. Furthermore, CDKN2A is associated with poor prognosis in various cancers, including colorectal cancer, yet the underlying causes remain elusive [9]. How CDKN2A regulates cuproptosis and tumor progression is still awaiting further in-depth research.

In this study, we conducted a comprehensive investigation into the expression, distribution, epigenetic modifications, regulation, and function of CDKN2A in colorectal cancer using an integrative approach combining single-cell sequencing, spatial transcriptomics, pathological image analysis, TCGA multi-omics analysis, and experimental validation. Through our research, we predicted and verified the regulatory mechanisms of CDKN2A in transcription and post-transcriptional regulation. We also dissected the changes in energy metabolism, copper metabolism, and copper ion transportation patterns mediated by CDKN2A expression. Additionally, we explored the abnormal signal transduction pathways associated with CDKN2A imbalance and mapped the microenvironment niche of tumor regions with CDKN2A expression. Our results provide novel insights into the role of CDKN2A in cuproptosis resistance and tumor progression, which have important implications for developing more effective diagnostic and therapeutic strategies for colorectal cancer.

## MATERIALS AND METHODS

### Processing and analysis of scRNA-seq data in colorectal cancer

The scRNA-seq data on colorectal cancer were obtained from the Gene Expression Omnibus (GEO) database under accession code GSE132465 [10]. The Seurat package was used to process the scRNA-seq data [11]. Filtering was performed by retaining cells that had unique molecular identifiers greater than 1000, expressing 200 to 6000 genes, and had mitochondrial content less than 20%. The count data were normalized using the “LogNormalize” method with a scale factor of 10,000. Genes with normalized expression between 0.125 and 3 and exceeding the 0.5 quantile of variance were identified as highly variable genes. Principal component analysis was conducted based on the highly variable genes. Multiple samples were integrated by the Harmony R package [12]. Dimensionality reduction and clustering were performed using the RunPCA, RunUMAP, FindNeighbors and FindClusters functions. When conducting cell labeling artificially, reference was made to marker genes used in past research [10, 13]. Cell trajectory inference was performed using Monocle 2 with DDR-Tree and the default parameters [14]. CytoTrace evaluated the pluripotent score for each cell fate [15]. Pseudobulk analysis was performed using the Libra package, with DESeq2 as the differential expression method and the Wald test for significance [16].

### Pathological image recognition

Immunohistochemically stained samples of colorectal cancer and normal colon were obtained from the HPA database [17]. To minimize the impact of poor slide quality on the results, one unanalyzable slide with severe wrinkles was excluded. The remaining 75 slides were analyzed using Qupath [18], implementing average channel, thresholds of 240, and sigma of 2.5 as parameters to determine tissue boundaries and assess tissue area. Positive staining was determined using the DAB channel with a threshold of 0.3 and sigma of 0.1. Subsequently, the sections underwent cell detection and segmentation, positive cell selection, and cell classification.

### Transcription factor inference and regulation network reconstruction

Regulons were computed using the single-cell regulatory network inference and clustering (pySCENIC) algorithm [19]. The motif data used for analyses were sourced from the cisTarget Human motif database v9. The limma R package was used to perform differential analysis on the transcription factor activity score matrix [20]. Regulators with |log2 (fold change)| > 0.5 and false discovery rate (FDR) < 0.05 were considered significantly differentially activated. The miRcode database was used to predict competitive endogenous RNA (ceRNA) networks [21]. The differentially expressed genes between tumor samples and normal samples were screened under |log2 (fold change)| > 1 and adjusted p-value < 0.05. Upregulated lncRNAs and downregulated miRNAs were selected as ceRNA members. Pearson correlation analysis between lncRNA and cuproptosis mRNA expression was significant at *P* < 0.05.

### Processing and analysis of scATAC-seq data

Publicly available scATAC-seq data on colorectal cancer were obtained from GEO under accession GSE201349 [22]. Pathologically confirmed colorectal samples and samples that were not affected by tumor invasion were included in the analysis. For donors with multiple replicates, one sample was randomly selected using the sample function in R. The ArchR package in R was used for quality control, doublet inference and removal (DoubletScores, filterDoublets functions), dimensionality reduction (addIterativeLSI function), batch correction (addHarmony function), gene score calculation (addGeneScoreMatrix function), clustering (addClusters function), visualization of clustering (addUMAP function), and marker feature (getMarkerFeatures function) identification [23]. Chromatin accessibility peaks were then called with Macs2 via ArchR with the addGroupCoverage, addReproduciblePeakSet and addPeakMatrix functions. Differential peaks were identified using the getMarkerFeatures function with |log2 (fold change)| > 1 and FDR < 0.05 as determined by Wilcoxon pairwise comparisons.

### Cell culture

Human colorectal cancer cell lines NCM460, HCT116, HT-29, COLO205, LOVO, SW620, SW480, and Caco2 were obtained from the Cell Bank of the Type Culture Collection Committee, Chinese Academy of Sciences (Shanghai, China), and the cell lines were cultured for a maximum of ten passages. Cells were cultured in Dulbecco’s Modified Eagle Media supplemented with 10% fetal bovine serum, 100 U/mL penicillin, and 100 μg/mL streptomycin at 37° C in a humidified 5% CO_2_ atmosphere.

### siRNA and mimic transfection

Small interfering RNAs (siRNAs) targeting SNHG7 (si-SNHG7) and CDKN2A (si-CDKN2A) were synthesized by General Pharma (Suzhou, China). The siRNA sequences utilized were as follows: siSNHG7 sense: 5′-GGCCUGACUACUUGCAATT-3′, siSNHG7 antisense: 5′-UUGCAAGAAUGUCAGGCCTT-3′; siCDKN2A sense: 5′-GCCACGCACCGAAUAGUTT-3′, siCDKN2A antisense: 5′-ACUAUUCGGUGCGUUGGGCTT-3′. Additionally, miR-133b mimics, negative control mimics, miR-133b inhibitor, and negative control inhibitor were procured from General Pharma, Shanghai, China, with the following sequences: miR-133b mimics sense: 5`-UUUGGUCCCCUUCAACCAGCUA-3′, miR-133b mimics antisense: 5′-GCUGGUUGAAGGGGCAAAUU-3′; miR-1336 inhibitor sense: 5′-UAGCUGGUUGAAGGGGACCAAA-3′, miR-133b inhibitor negative control sense: 5’-CAGUACUUUUGUGUAGUACAA-3′; U6 sense 5’-CTCGCTTCGGCAGCACA-3’, U6 sense antisense 5’-AACGCTTCACGAATTTGCGT-3’ (Supplementary Table 2). Cells were transfected with 50 nM siRNA or 200 nM miRNA mimic and inhibitor using TurboFect Transfection Reagent (Thermo Fisher Scientific, Shanghai, China) according to the manufacturer’s protocol. Transfected cells were harvested 24-48 hours later for subsequent experiments.

### Western blotting

Cells were lysed in RIPA buffer, and the resultant supernatant was collected for protein concentration determination using the BCA protein assay kit (P5026, Sangon, Shanghai, China). Subsequently, 20 μg of protein was loaded onto an 8%-15% SDS-PAGE gel. The separated proteins were transferred onto a PVDF membrane (Millipore, Carrigtwohill, Ireland) for 90 min. The membrane was then blocked with TBST buffer (Tris-buffered saline with 0.1% Tween 20) containing 5% milk powder for 2 h, followed by overnight incubation with the primary antibodies (FDX1: AB108257, GAPDH: YM3029). Immunoblotting was carried out using secondary antibodies and the ultra-high sensitivity ECL kit (HY-K1005, MCE, Shanghai, China), with visualization of resulting bands performed using a ChemiScope 6100 Imager (QinXiang Products Ltd., Shanghai, China).

### Cell viability assessment

Cell viability was evaluated utilizing the Cell Counting Kit-8 (CCK-8) (B34304, Selleck, Shanghai, China). Specifically, 5 × 10^3^ cells were seeded in individual wells of a 96-well plate and exposed to designated treatments at predetermined intervals. Subsequently, 10 μL of CCK-8 solution was introduced into each well, followed by an incubation period at 37° C for 45 minutes to 1 h. Absorbance at 450 nm was measured using a microplate reader (Synergy 2, Bio-Tek Instruments, Winooski, VT, USA). Each experimental condition was assessed in 4 to 6 replicate wells, and each experiment was performed independently at least triple. Absorbance readings were normalized to the negative control (NC) group, and graphical representations were constructed based on the average values derived from two separate experimental runs.

### Transwell migration assay

Cells were diluted in serum-free 1640 medium to a concentration of 5 × 10^4^ cells/well and seeded into the upper chamber of a Transwell insert. The lower chamber was supplemented with 10% FBS 1640 medium to serve as a chemoattractant. Following a 48 h incubation period, cells were fixed with 75% paraformaldehyde and stained with 0.1% crystal violet. Migration quantification was performed using ImageJ software (version 1.53v).

### RNA extraction and qRT-PCR

Total RNA was extracted from cells using TRIzol reagent (Invitrogen, Waltham, MA, USA) according to the manufacturer’s instructions. RNA was reverse transcribed into cDNA using the PrimeScript RT Reagent Kit (Takara, Beijing, China). Quantitative real-time PCR was performed in triplicate using SYBR Premix Ex Taq II (Takara, Beijing, China) on a Bio-Rad CFX96 Touch Real-Time PCR Detection System. Target gene expression was normalized to GAPDH. miR-133b expression was normalized to U6 snRNA. Relative expression was calculated by the 2^−ΔΔCt^ method. Primers are listed in Supplementary Table 1.

### Intracellular Cu^2+^ measurement

Intracellular Cu^2+^ levels were measured using a copper colorimetric detection kit (Elabscience, Wuhan, China) according to the manufacturer’s instructions. HCT116 and HT-29 cells were collected, rinsed with PBS, and lysed. Cell lysates were mixed with the copper reaction solution and incubated for 30 min at room temperature. Absorbance at 580 nm was measured using a microplate reader. Intracellular copper concentrations were calculated based on a standard curve and normalized to total protein content.

### Differential analysis of colorectal cancer cell line RNA-seq data

RNA-seq data for colorectal cancer cell lines were obtained from the GEO database (accession GSE153412) [24]. Expression data were log2 transformed, batch corrected and differentially analyzed using the limma package. Differentially expressed genes were defined as having |log2FC| > 1 and an adjusted p-value < 0.05.

### Inference and annotation of malignant regions in spatial transcriptome samples

Four spatial transcriptomic samples of colorectal cancer and liver metastases were obtained from the Single-Cell Colorectal Cancer Liver Metastases database and named colon1, colon2, lm1, and lm2 [25]. Samples were normalized separately using SCTransform. The SpaCET.deconvolution function from the spaCET package was used to calculate the malignancy score for spots [26]. Hematoxylin and eosin (H&E)-stained sections were segmented using QuPath to characterize the tumor and normal regions [18]. To accurately annotate spots, we considered the following: 1) Seurat clustering, 2) marker expression, 3) SpaCET malignancy scores, 4) H&E staining, and 5) pathological review. Finally, the spot annotation was manually adjusted using our developed package SpotSweeper. In tumor regions, spots were defined as CDKN2A^+^ spots, CDKN2A^-^ spots, or others (necrotic region). In nontumor regions, spots were classified as nearby CDKN2A^+^, nearby CDKN2A^-^, nearby none, or nearby both based on proximity to CDKN2A^+^ or CDKN2A^-^ spots.

### Immunoinfiltration assessment and spatial communication analysis

Gene signatures of myeloid cells and T cells in colorectal cancer were obtained from published scRNA-seq studies [27]. Immune infiltration levels were evaluated by ssGSEA. Differential enrichment was defined as the ratio of median enrichment scores between groups [28]. Tumor transcriptional diversity was calculated as the median absolute deviation of Pearson correlations between highly variable genes in tumor regions multiplied by 1.4826 [29]. CellChat was used to infer communication networks between tumor subregions [30].

### TCGA multiomics data analysis

TCGAbiolinks retrieved colorectal cancer data from TCGA, including RNA-seq, DNA methylation, CNV, mutations, and clinical data [31]. ATAC-seq data were obtained from the NIH Genomic Data Commons (https://gdc.cancer.gov/about-data/publications/ATACseq-AWG). Differential expression analysis was performed with DESeq2 (|log2 (fold change)| > 1 and adjusted p-value < 0.05) [32]. Differential methylation sites were identified using TCGAanalyze_DMC. GISTIC 2.0 was used to analyze CNVs on GenePattern (https://www.genepattern.org/, confidence=0.99) [33]. Maftools was used to compare mutation frequencies between groups (mafCompare function, p-value < 0.05).

### Enrichment analysis and gene set variation analysis

KEGG pathway enrichment was performed with the enrichKEGG function in the clusterProfiler package [34]. Reactome pathways were enriched using the enrichPathway function in ReactomePA [35]. Gene set variation analysis utilized the GSVA package [36]. Metabolic gene sets were retrieved from published studies [25, 37]. EMT-related gene sets were extracted from the EMTome platform ([38], criteria: colorectal tissue source, human species, RNA-seq/microarray methods). Limma compared pathway activity scores between CDKN2A^+^ and CDKN2A^-^ epithelial cells.

### Statistical analysis

The statistical analysis methods and standards for sequencing data were as described above, and experimental data analysis was performed using SPSS v20. Pearson correlations were used to test the relationships between variables. Group comparisons utilized Student’s t-tests, Wilcoxon signed-rank tests, or one-way ANOVA as appropriate. *P* < 0.05 indicated statistical significance.

### Availability of data and materials

Our analysis pipeline for spatial transcriptomics was developed into an R package, SpotSweeper (https://github.com/Biocxifu/SpotSweeper), enabling convenient manual annotation of spots, visualization, selection of adjacent spots based on specific tumor groups, immune infiltration analysis, evaluation of sample transcriptome heterogeneity, and analysis of spatial communication.

### Consent for publication

The authors give their consent for the publication of the manuscript in *Aging*.

## RESULTS

### Transformation of cuproptosis characteristics during tumor evolution

**Figure 1:**
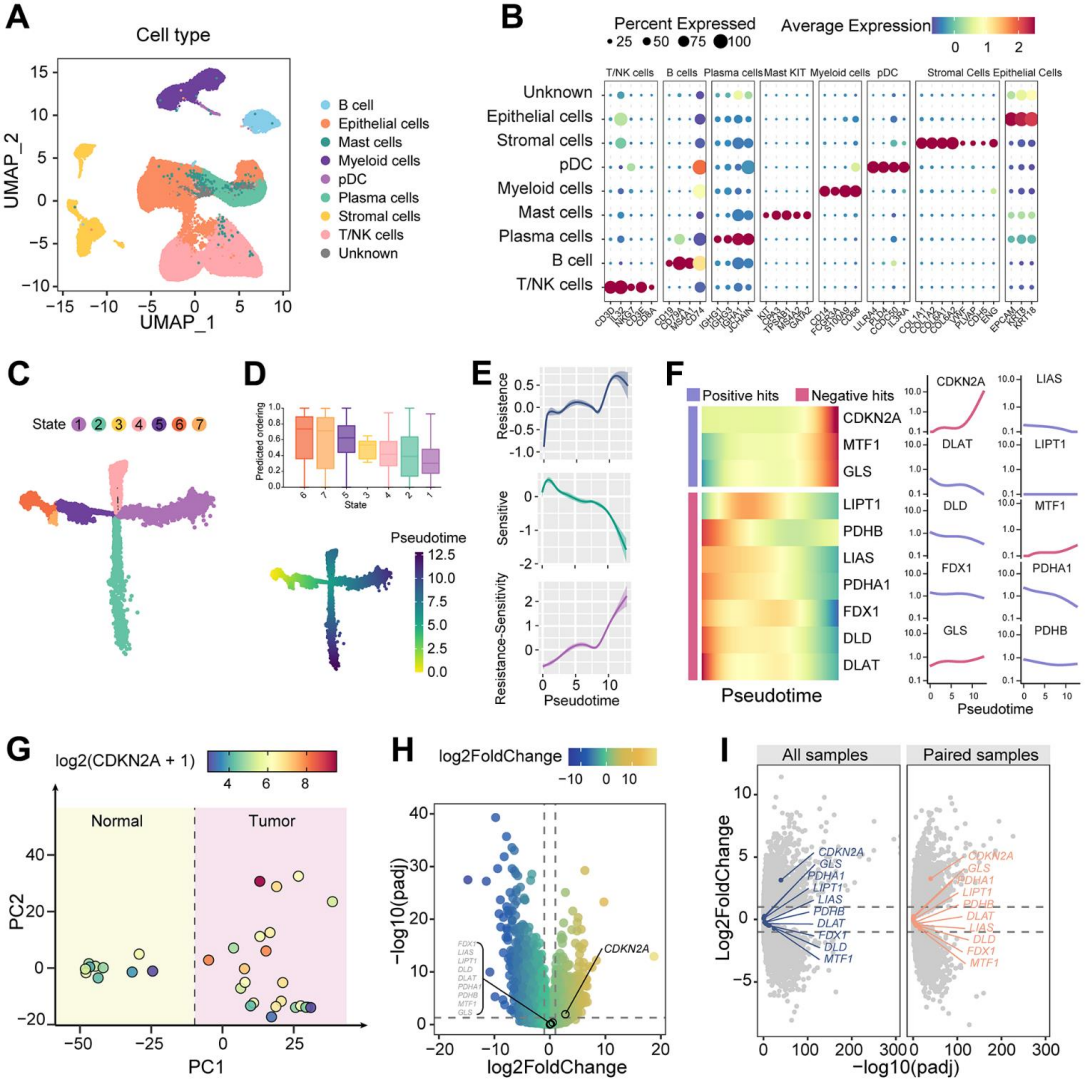
**Dynamic alterations of cuproptosis characteristics.** (**A**) UMAP dimension reduction plots showing cell clusters identified by scRNAseq. (**B**) Expression of marker genes of different cell clusters. (**C**) Evolution trajectory of tumor epithelial cells, which are divided into seven states. (**D**) TOP: CytoTRACE predicts the evolutionary stage of each state. Bottom: pseudotime in the evolutionary trajectory. (**E**) Shaded line plot indicating the resistance score, sensitivity score, and the difference between the two for cuproptosis in tumor epithelium along pseudotime. (**F**) Heatmap and line plot showing cuproptosis-related genes arranged in pseudotime. (**G**) PCA map of the cell count matrix obtained from pseudobulk analysis. (**H**) Differential gene expression analysis of genes obtained from pseudobulk analysis. (**I**) Differential gene analysis between tumor and normal samples, as well as between tumor and paired samples, using the TCGA colorectal cancer cohort.

To investigate the expression characteristics of cuproptosis-associated genes in colorectal cancer, we performed dimensionality reduction clustering on scRNAseq data from colorectal cancer and normal tissues. Based on the expression levels of marker genes for different cell types, we identified epithelial cells, stromal cells, immune cells, and a group of unknown cells (Figure 1A, 1B). In the evolutionary trajectory constructed on tumor epithelial cells (Figure 1C), cytoTRACE identifies state 6 as the earliest pseudotime cell (Figure 1D). It is noteworthy that the resistance capacity of tumor epithelial cells to cuproptosis is increasing along the trajectory of tumor evolution (Figure 1E). Genes associated with cuproptosis resistance showed a general increasing trend, while the expression of sensitive genes decreased. Among them, the increasing trend of CDKN2A was the most representative (Figure 1F). This indicates the acquisition of cuproptosis resistance phenotype during malignant tumor progression.

Furthermore, we focused on the molecular characteristic changes associated with cuproptosis in tumor formation. After pseudobulk processing of epithelial cells, principal component analysis (PCA) clustering showed good differentiation between tumor and normal epithelial cells (Figure 1G). Differential expression analysis revealed that CDKN2A was highly expressed in tumor epithelial cells compared to normal intestinal epithelial cells, while other cuproptosis-related genes were unchanged (Figure 1H). Validation using TCGA data confirmed elevated CDKN2A but not other cuproptosis-related genes in tumors versus normal colon tissue (Figure 1I). These findings highlight the central role of aberrantly high expression of CDKN2A in both tumor progression and cuproptosis resistance in colorectal cancer.

### CDKN2A exhibits heterogeneous distribution in colorectal cancer

**Figure 2:**
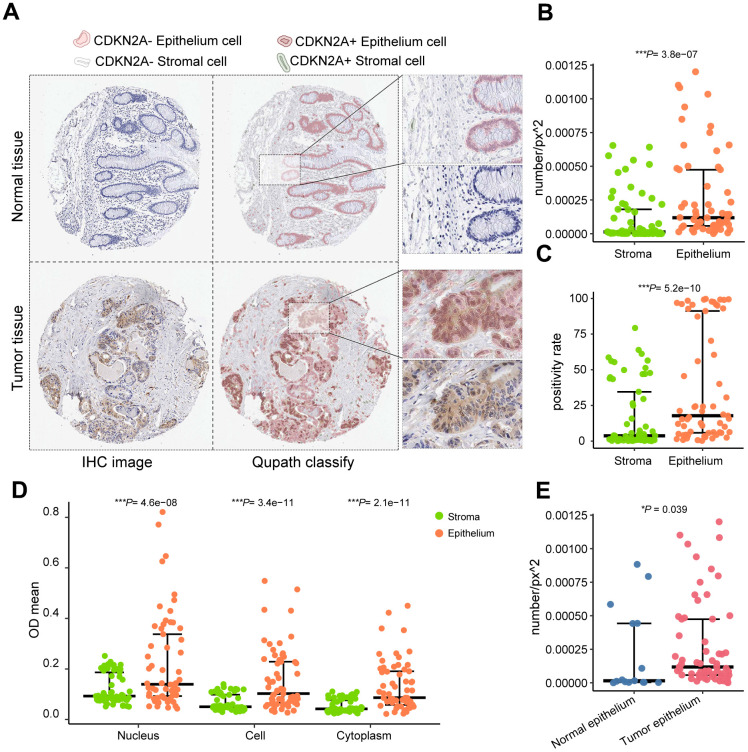
**QuPath performs cell identification on immunohistochemical staining images from the Human Protein Atlas.** (**A**) Identified CDKN2A-positive cells and cell types. (**B**) Comparison of CDKN2A-positive expression density between epithelial tissue and stroma. (**C**) Comparison of the CDKN2A-positive expression rate between epithelial tissue and stroma. (**D**) Comparison of CDKN2A-positive staining intensity between epithelial tissue and stroma. (**E**) Comparison of the positive density of CDKN2A between epithelial tissue in tumor and normal control samples using the Wilcoxon test. ***P < 0.001.

To visualize the expression and distribution of CDKN2A in tissues, we employed Qupath for image recognition on immunohistochemical sections. Epithelial cells and stromal cells of both colorectal cancer and normal tissue slices were identified (Figure 2A). Significantly higher positive rates, OD values, and positive densities of CDKN2A were observed in tumor epithelial cells compared to stromal cells (Figure 2B–2D). Tumor epithelium displayed a higher density of CDKN2A positivity in comparison with normal tissue (Figure 2E). These distributional patterns suggest localized and heterogeneous overexpression of CDKN2A in the epithelium of colorectal cancer.

### Regulatory mechanisms underlying CDKN2A upregulation

**Figure 3:**
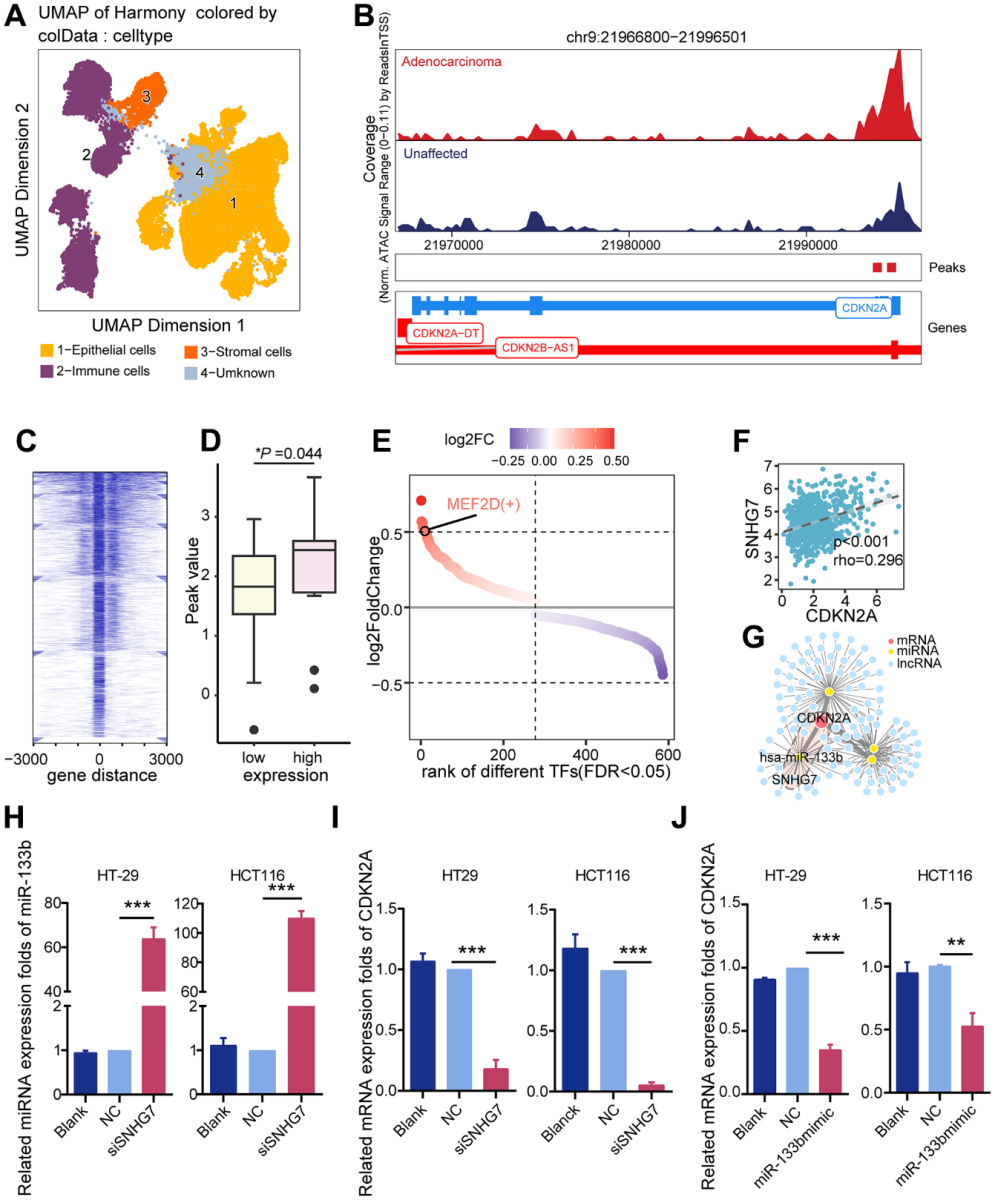
**Potential regulatory mechanisms of CDKN2A.** (**A**) UMAP dimension reduction plots showing annotated cell clusters, with different colors representing different cell types. (**B**) Genome accessibility trajectory around CDKN2A in tumor epithelium and normal epithelium cells, with peaks called in the scATAC data and peaks-to-gene links indicated below the tracks. (**C**) TSS distribution heatmap for the ATAC-seq data in the TCGA colorectal cancer cohort. (**D**) Peak value differences in CDKN2A sites between the CDKN2A high and low expression groups in the TCGA colorectal cancer cohort, *P < 0.05, Wilcoxon test. (**E**) Transcription factors predicted by Pyscenic to potentially regulate CDKN2A, ranked based on the fold difference in AUCell values for each point. (**F**) A ceRNA regulatory network was constructed based on differentially expressed genes, with the SNHG7/miR-133b/CDKN2A regulatory axis being the most relevant potential regulatory network. (**G**) Scatter plot showing the CDKN2A and SNHG7 expression levels, the Spearman rank test. (**H**) qRT-PCR analysis was performed to assess the influence of silencing SNHG7 on miR-133b expression levels in CRC cell lines. (**I**) qRT-PCR analysis was conducted to investigate the influence of SNHG7 knockout on CDKN2A expression levels in CRC cell lines. (**J**) qRT-PCR analysis was employed to determine the effects of miR-133b mimic and miR-133b inhibitor transfection on CDKN2A expression levels in CRC cell lines.

To elucidate potential mechanisms driving CDKN2A upregulation in colorectal cancer, we examined epigenetic modification and transcriptional regulation. ScATACseq data was analyzed using ArchR to identify epithelial cells derived from both colorectal cancer and unaffected tissues (Figure 3A). Higher chromatin accessibility levels were observed in colorectal cancer epithelial cells compared to normal epithelial cells within the CDKN2A gene region (Figure 3B). Similar findings were obtained in the TCGA cohort. A significant increase in chromatin accessibility was observed at the CDKN2A gene locus in tissues exhibiting elevated CDKN2A expression levels (Figure 3C, 3D). Nonetheless, no statistically significant correlation was observed between CDKN2A expression and either copy number variation or promoter methylation (Supplementary Figure 1). Leveraging the observed changes in chromatin accessibility, pySCENIC was employed to identify transcription factors capable of regulating the transcription of CDKN2A. The results show that MEF2D is enriched in CDKN2A-expressing tumor epithelia and can target CDKN2A (Figure 3E).

Besides investigating epigenomic and genomic factors, we constructed a competing endogenous RNA (ceRNA) network to explore post-transcriptional regulatory factors that influence CDKN2A expression. Long non-coding RNAs (lncRNAs) have the ability to act as ceRNAs to regulate target genes. Based the miRcode database, we constructed a regulatory network consisting of lncRNAs, miRNAs, and mRNAs that are involved in the regulation of CDKN2A in the TCGA cohort (Figure 3F). Particularly noteworthy, the expression of SNHG7 exhibited the strongest correlation with CDKN2A (rho = 0.296, *P* < 0.001; Figure 3G). These findings were validated in both colorectal cancer tissues and cell lines, confirming the upregulation of CDKN2A and SNHG7 along with the downregulation of miR-133b (Supplementary Figure 2). Knockdown of SNHG7 increased miR-133b and decreased CDKN2A expression (Figure 3H, 3I). miR-133b mimic transfection decreased CDKN2A expression, and miR-133b inhibitor transfection increased CDKN2A expression (Figure 3J). Our findings suggest the existence of an SNHG7/miR-133b/CDKN2A network that potentially acts to inhibit cuproptosis in colorectal cancer.

### CDKN2A enhances glycolysis and regulates copper homeostasis

**Figure 4:**
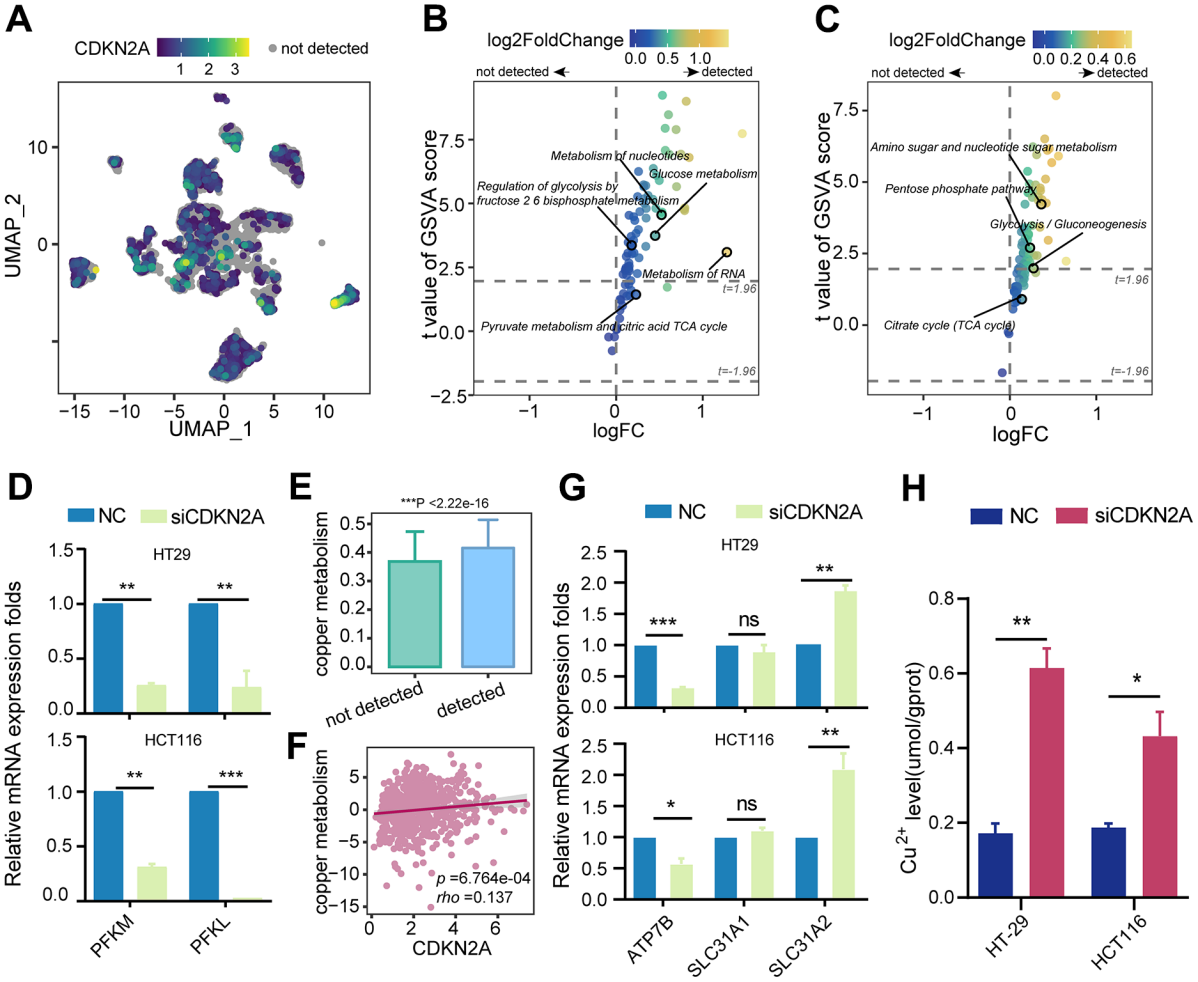
**The impact of CDKN2A on energy metabolism, copper metabolism, and copper ion transportation.** (**A**) UMAP dimension reduction plots of colorectal cancer epithelium cells showing the expression level of CDKN2A, with color depth representing the level of CDKN2A expression and gray indicating undetected CDKN2A expression. (**B**) Reactome metabolic pathways for different CDKN2A expression levels in tumor epithelium cells. (**C**) KEGG metabolic pathways for different CDKN2A expression levels in tumor epithelium cells. (**D**) Knockdown of CDKN2A leads to a decrease in the mRNA expression levels of phosphofructokinase-1 genes (PFKM, PFKL) in CRC cell lines. (**E**) Comparison of copper metabolism scores among different CDKN2A expression groups in tumor epithelial cells. ***P < 0.001, Wilcoxon test. (**F**) A scatter plot was generated to illustrate the correlation between CDKN2A expression and copper metabolism in the TCGA cohort. (**G**) qRT-PCR was utilized to examine the impact of CDKN2A knockdown on the mRNA transcriptional levels of copper transporters (SLC31A1, SLC31A2, ATP7B) in CRC cell lines. (**H**) Knocking out CDKN2A increases the intracellular copper ion concentration. ns represents no significance; *P < 0.05, **P < 0.01, ***P < 0.0001.

Considering the close correlation between cuproptosis, cellular metabolism, and copper ion overload, we propose that CDKN2A is involved in regulating abnormal metabolic patterns and copper ion transport in tumors. Therefore, we initially assessed the KEGG and Reactome metabolic pathways in tumor epithelial cells. In the Reactome metabolic pathway terms, epithelial cells expressing CDKN2A showed higher activity in glucose metabolism and regulation of glycolysis by the fructose 2,6-bisphosphate metabolism (Figure 4A, 4B). Similarly, the activity of the glycolysis pathway was significantly upregulated in the KEGG metabolic pathway terms (Figure 4C). Although no significant changes were found in the TCA cycle pathway directly associated with cuproptosis, the identified upregulation of the glycolysis pathway may be associated with CDKN2A-mediated resistance to cuproptosis [39]. Fructose-2,6-bisphosphate can activate phosphofructokinase-1, the rate-limiting enzyme in glycolysis that influences the flux of glycolysis [40]. Further experiments revealed that silencing CDKN2A markedly decreased the mRNA levels of the phosphofructokinase genes PFKL and PFKM in colorectal cancer cell lines (Figure 4D). This confirms that CDKN2A can modulate the intensity of glycolysis by impacting the levels of phosphofructokinase, thereby resisting cuproptosis.

In parallel, tumor epithelial cells expressing CDKN2A displayed increased activity in copper metabolism (Figure 4E), and this finding was corroborated by correlation analysis in the TCGA cohort (Figure 4F). Following CDKN2A knockout, we detected an upregulation of the copper uptake gene SLC31A2 and a downregulation of the copper efflux gene ATP7B in colorectal cancer cell lines (Figure 4G). Moreover, intracellular Cu^2+^ levels in colorectal cancer cell lines exhibited a significant increase (Figure 4H). These findings provide evidence that CDKN2A modulates copper metabolism and copper ion concentration to confer cuproptosis resistance.

### CDKN2A-positive tumor epithelial cells exhibit high Wnt pathway activity and a tendency towards EMT phenotype

**Figure 5:**
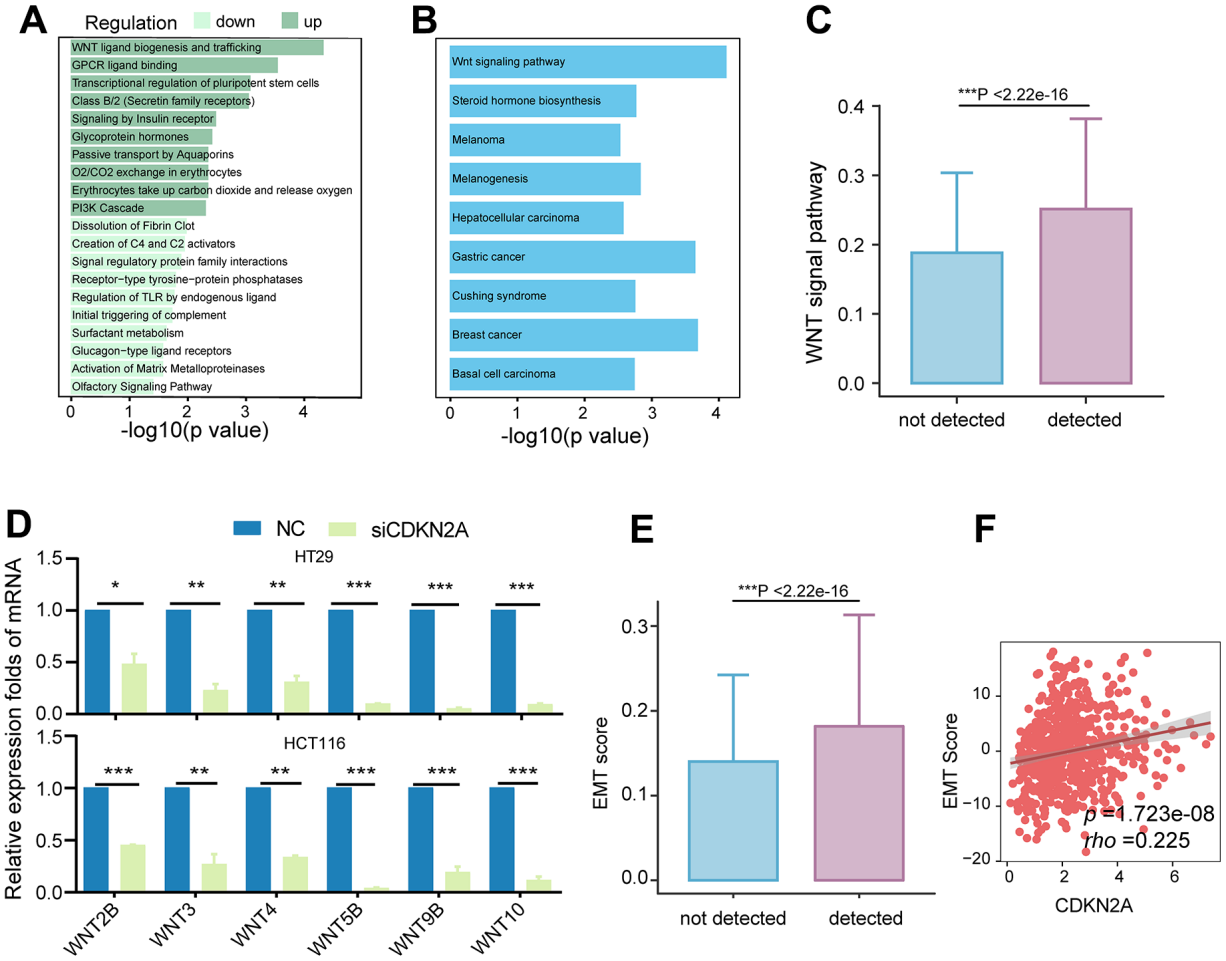
**Association of CDKN2A with the Wnt signaling pathway and EMT.** (**A**) Enriched Reactome pathways for differentially expressed genes in the TCGA colorectal cancer cohort, with dark green representing pathways involving upregulated genes and light green representing pathways involving downregulated genes. (**B**) KEGG pathways enriched for upregulated genes in the CDKN2A high-expression group, showing upregulation of the Wnt signaling pathway. (**C**) Comparison of Wnt signaling pathway scores between different CDKN2A expression groups in tumor epithelium cells, ***P < 0.001, Wilcoxon test. (**D**) Knockdown of CDKN2A leads to decreased mRNA expression levels of Wnt signaling pathway members in CRC cell lines. *P < 0.05, **P < 0.01, ***P < 0.0001. (**E**) Comparison of EMT scores among different CDKN2A expression groups in tumor epithelial cells. ***P < 0.001, Wilcoxon test. (**F**) The scatter plot shows the correlation between the expression level of CDKN2A and EMT score in the TCGA cohort.

We further explored the relationships between CDKN2A and nonmetabolic pathways. The samples in the TCGA cohort were categorized into high and low CDKN2A groups based on their average expression levels. The upregulated genes in the high CDKN2A group were mainly involved in the biogenesis and transport of Wnt ligands (Figure 5A). Moreover, KEGG enrichment analysis demonstrated the associations between upregulated genes and the Wnt signaling pathway (Figure 5B). Similarly, tumor epithelial cells expressing CDKN2A exhibited elevated Wnt pathway activity (Figure 5C). CDKN2A knockdown significantly downregulated Wnt genes in colorectal cancer cell lines (Figure 5D). These findings provide strong evidence that CDKN2A promotes colorectal cancer through the activation of Wnt signaling.

Given prior evidence of CDKN2A EMT involvement [41] and the established role of Wnt signaling in EMT [42, 43], we hypothesize that CDKN2A expression influences the EMT process. Consequently, tumor epithelial cells expressing CDKN2A exhibited elevated EMT scores (Figure 5D). Furthermore, this observation was validated in the TCGA cohort, confirming a positive correlation between CDKN2A expression and EMT (Figure 5E, 5F). These findings suggest that aberrant CDKN2A overexpression enhances Wnt signaling, thereby influencing metabolism, progression, and conferring resistance to cuproptosis.

### High expression of CDKN2A characterizes high genomic instability and sensitivity to radiation and chemotherapy

**Figure 6:**
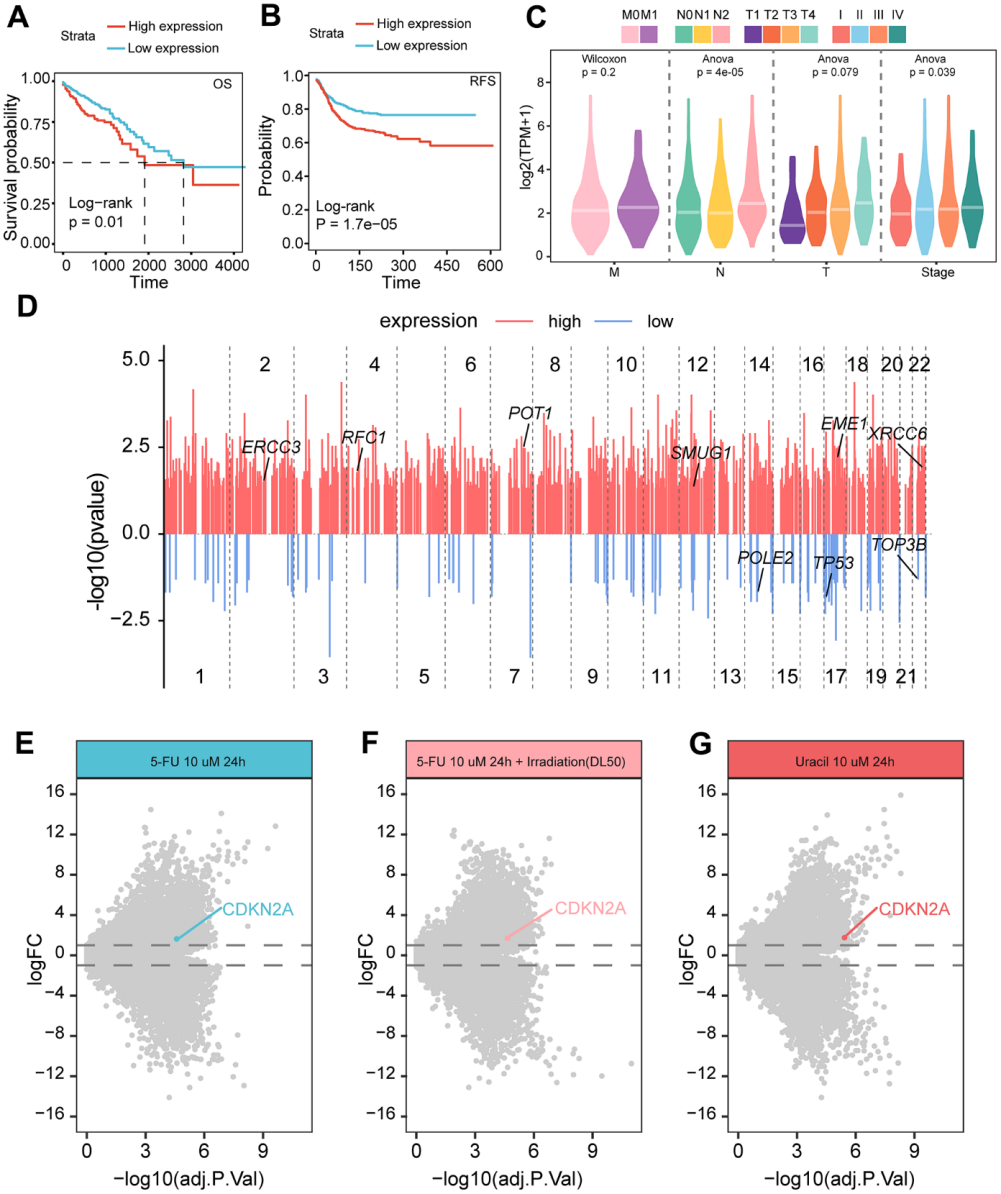
**Relationship between CDKN2A expression and prognosis and treatment of colorectal cancer.** (**A**) The Kaplan-Meier curve demonstrates a correlation between high CDKN2A expression and decreased overall survival rates in colorectal cancer patients. (**B**) The Kaplan-Meier curve illustrates that high CDKN2A expression is linked to reduced disease-free survival in colorectal cancer patients. (**C**) The association between CDKN2A and clinical pathological features, including the TNM staging and pathological stage of the tumor. (**D**) The differences in mutated genes between CDKN2A expression groups are shown, with red lines representing genes with increased mutation rates in the high expression group, and blue lines representing genes with increased mutation rates in the low expression group. The annotated genes are involved in DNA damage repair. The X-axis represents the genomic position coordinates, while the Y-axis represents the negative logarithm of the P-values for rate comparison. (**E**) Genes differentially expressed between 5-FU-S and 5-FU-R cell lines after 5-fluorouracil treatment. (**F**) Genes differentially expressed between 5-FU-S and 5-FU-R cell lines after 5-fluorouracil plus radiation therapy. (**G**) Genes differentially expressed between 5-FU-S and 5-FU-R cell lines after uracil treatment.

In order to investigate the relationship between the clinical application value of CDKN2A in colorectal cancer, we conducted survival analysis and found that patients with high expression of CDKN2A had worse survival prognosis (Figure 6A, 6B). Clinical pathological parameters showed that CDKN2A was correlated with higher T, N staging, and pathological staging (Figure 6C), indicating that CDKN2A can serve as a biomarker for clinical prognosis. Given the previously observed heightened activity in nucleotide metabolism (Figure 4B, 4C), we inferred that the expression of CDKN2A can also serve as an indicator of genomic alterations. Therefore, we compared the gene mutations between the high and low CDKN2A expression groups and found that the genomes of the high expression group exhibited extensive mutations, including some genes involved in DNA damage repair (Figure 6D). Furthermore, in the 5-fluorouracil-resistant (5-FU-R) and 5-fluorouracil-sensitive (5-FU-S) cell lines screened after drug resistance induction, we found that the 5-FU-S group exhibited high levels of CDKN2A expression during treatment with 5-fluorouracil, 5-fluorouracil plus radiation therapy, and uracil (Figure 6E–6G). This indicates that the high expression of CDKN2A may suggest increased sensitivity to chemotherapy and the combination of radiation therapy and chemotherapy.

### Spatial transcriptome analysis reveals CDKN2A expression patterns in colorectal cancer

**Figure 7:**
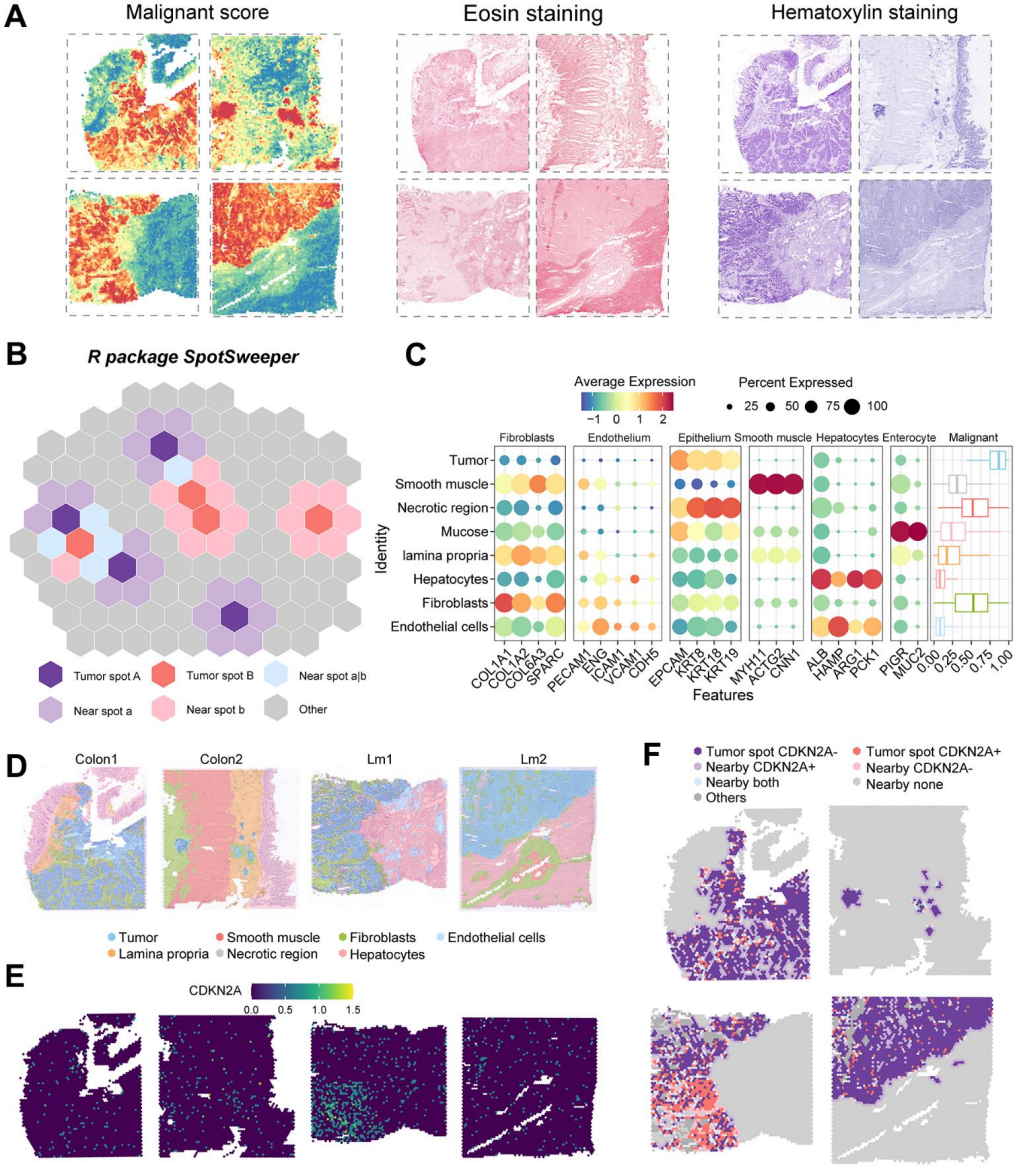
**Regional annotation and expression of CDKN2A in spatial transcriptomic samples.** (**A**) Malignancy scores on colorectal cancer spatial transcriptomic samples and on two types of tumor tissue stained with H&E. (**B**) Analysis of colorectal cancer spatial transcriptomic samples using the SpotSweeper R package. (**C**) Marker gene expression of all cell types in the tissues. Color depth represents the average expression level, and dot size represents the percentage of expressing cells. (**D**) Annotation of results from all sample regions, with different colors representing different regions. Sample numbers are listed above. (**E**) Expression of CDKN2A in colorectal cancer tissues. (**F**) Expression of CDKN2A in tumor regions and adjacent regions.

To visually examine the spatial expression pattern of CDKN2A in colorectal cancer, we analyzed samples using spatial transcriptomics. By utilizing malignancy scoring, H&E staining of the slides (Figure 7A), Seurat clustering, marker expression, pathologist identification, and the SpotSweeper package for accurate annotation correction (Figure 7B), we identified the following regions: tumor, smooth muscle, fibroblasts, endothelial cells, lamina propria, necrotic region, and hepatocytes (Figure 7C, 7D). To avoid analytical bias, necrotic tumor regions were excluded from further analysis. Within the tumor area, we designated spots as CDKN2A^+^ and CDKN2A^-^. In the non-tumor area, we identified nearby spots classified as CDKN2A^+^, CDKN2A^-^, neither, or both (Figure 7E, 7F). The spatial mapping demonstrated distinct patterns of CDKN2A expression in colorectal cancer.

### Microenvironment in CDKN2A-positive region exhibits pro-tumor ecological niche

**Figure 8:**
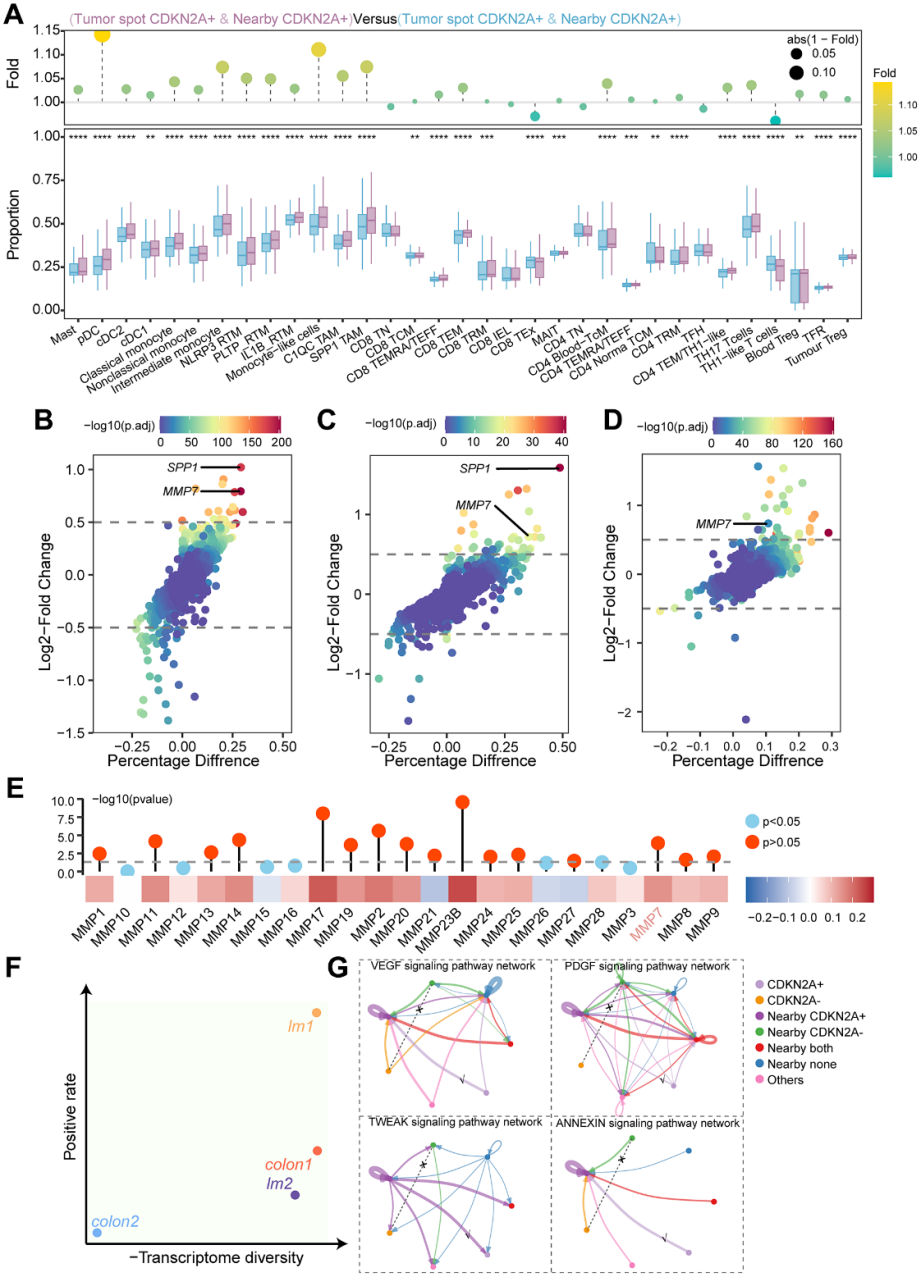
**Immune cell infiltration, differential gene expression, and cellular communication networks in tumor regions and adjacent regions.** (**A**) The ssGSEA algorithm evaluated the relationship between CDKN2A expression levels and immune cell infiltration levels in different regions of colorectal cancer. The lower box plot shows the difference in immune cell infiltration between the different regions, and the upper bar chart shows the fold change. Statistical significance was determined using the Wilcoxon test (***P < 0.001). (**B**) Within the TCGA cohort, high CDKN2A expression was associated with increased infiltration of SPP1 in TAMs. The Wilcoxon test was utilized for statistical analysis (**P < 0.01). (**C**) Differential gene analysis of CDKN2A^+^ tumor spots and CDKN2A- tumor spots in the spatial transcriptomics analysis. The x-axis represents the difference in the proportion of gene expression between the two groups, and the y-axis represents the fold change. (**D**) Differential gene analysis of nearby CDKN2A^+^ and nearby CDKN2A- regions in spatial transcriptomics analysis. (**E**) Differential gene analysis of tumor epithelial cells expressing CDKN2A and those not expressing CDKN2A in the scRNAseq cohort. (**F**) Heatmap showing the correlation between the expression levels of CDKN2A and MMPs in TCGA the cohort. The bar chart above represents the significance of P-values, with different colors indicating statistical significance. The statistical method used was the Spearman rank test. (**G**) Transcriptional heterogeneity and expression rate of CDKN2A in all samples, where the x-axis represents the inverse of the transcriptional heterogeneity score, and the y-axis represents the expression rate of CDKN2A. (**H**) Cell communication network diagram between tumor spots and adjacent spots.

To investigate the TME characteristics of the CDKN2A expression region, we used the ssGSEA algorithm to evaluate immune cell infiltration scores in the tissue sections. CDKN2A-expressing tumors and adjacent regions exhibited varying levels of immune infiltration. pDC, monocyte-like cells, and SPP1^+^ TAM were highest in the CDKN2A^+^ regions and adjacent regions, with SPP1^+^ TAMs linked to poor prognosis (Figure 8A) [27]. The TCGA cohort showed similar results (Figure 8B). MMP7 and SPP1 levels were also higher in both CDKN2A^+^ tumor regions and nearby CDKN2A^+^ regions (Figure 8C, 8D). We validated the results in the ScRNAseq cohort and detected the upregulation of MMP7 in CDKN2A-positive tumor cells (Figure 8E). The TCGA cohort showed similar results where a strong positive correlation existed between CDKN2A expression and various MMPs, including MMP7 (Figure 8F), indicating that CDKN2A can facilitate invasion and metastasis.

We examined the transcriptomic heterogeneity in both the tumor regions and the CDKN2A expression rates of each sample. In the low-heterogeneity sample “lm1”, CDKN2A exhibited high expression rate (Figure 8G). CellChat analysis of “lm1” revealed that CDKN2A^+^ tumors and the nearby regions communicated via VEGF, PDGF, ANNEXIN, and TWEAK signaling. However, no signal transmission was observed between the tumor spot CDKN2A^-^ and nearby CDKN2A^-^ cells in these signals (Figure 8H). This suggests that the microenvironment of the CDKN2A^+^ region is predisposed to immune suppression, angiogenesis, and tumor invasion. The discovered TME landscape can expand our understanding of how CDKN2A mediates resistance to cuproptosis and promotes tumor progression.

### CDKN2A regulates the viability and migration of CRC cells by inhibiting cuproptosis

**Figure 9:**
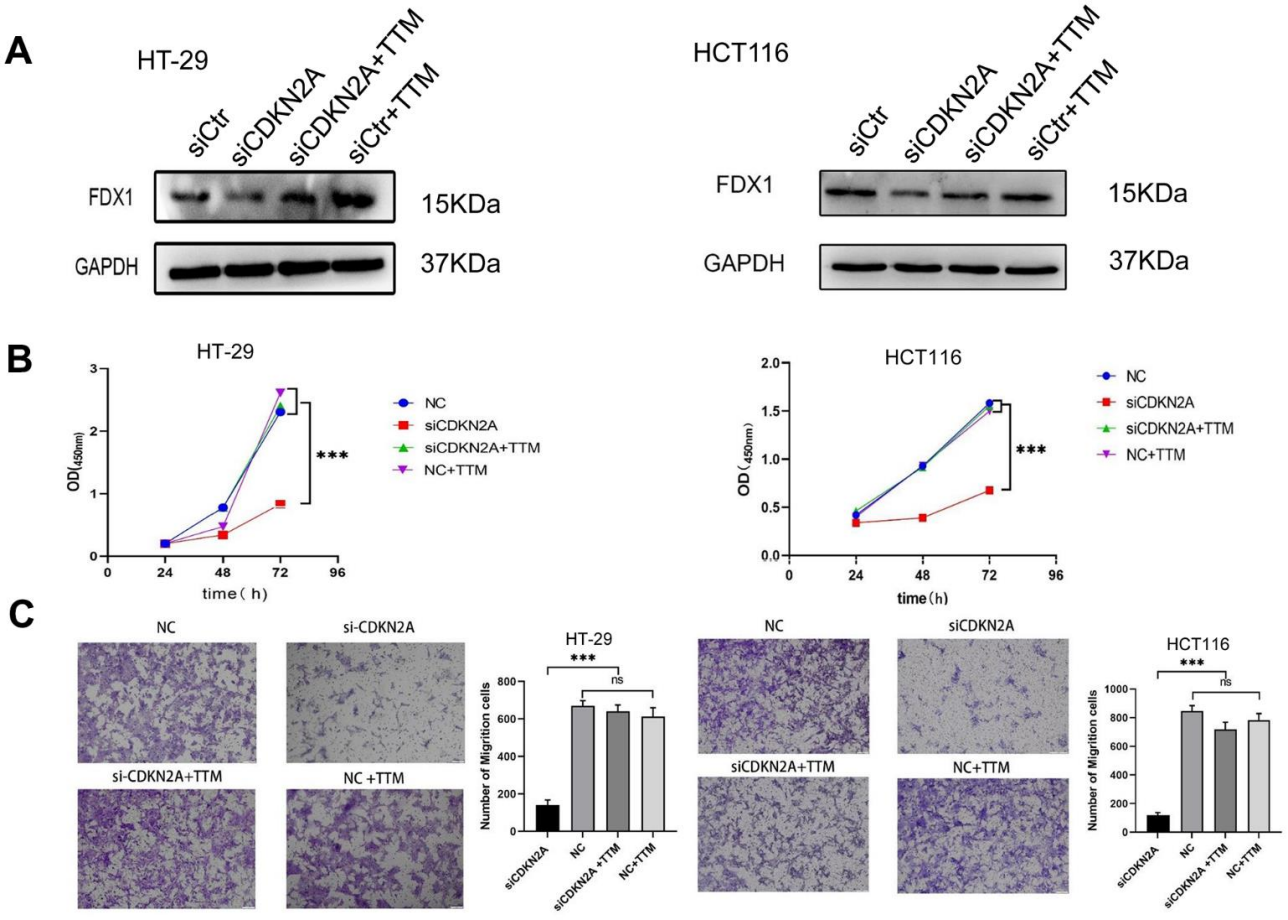
**CDKN2A enhances colorectal cancer cell viability and migration through suppressing cuproptosis.** (**A**) Western blot analysis was performed to examine the expression changes of FDX1 in HT-29 and HCT116 cells following knockdown of CDKN2A and addition of a cuproptosis inhibitor (*P<0.05, **P<0.01, ***P<0.001). (**B**) The CCK8 assay was utilized to assess alterations in cell proliferation ability in HT-29 and HCT116 cells upon CDKN2A knockdown and cuproptosis inhibitor treatment (*P <0.05, **P <0.01, ***P <0.001). (**C**) Transwell assay was employed to evaluate changes in cell invasion ability in HT-29 and HCT116 cell lines following CDKN2A knockdown and cuproptosis inhibitor supplementation (*P <0.05, ** *P* <0.01, *** *P* <0.001).

To assess the potential of CDKN2A in regulating CRC cell behavior through cuproptosis inhibition, we employed siRNA to silence CDKN2A expression in cells. As depicted in Figure 9A, cell viability was diminished, and the expression of iron-sulfur cluster protein FDX1 decreased in CDKN2A-knockdown HT-29 and HCT116 cells. Moreover, the attenuated CDKN2A expression led to reduced migratory capacity in HT-29 and HCT116 cells. However, upon supplementation with a cuproptosis inhibitor, cell proliferation and migration were restored, accompanied by a reversal in FDX1 expression (Figure 9B, 9C). These findings collectively indicate the pivotal role of CDKN2A in modulating the growth and migration of CRC cells through its interaction with cuproptosis.

## DISCUSSION

Previous pan-cancer study of cuproptosis-associated genes based TCGA data revealed universal upregulation of CDKN2A across 16 different cancer types, including colorectal cancer [44]. This aligns with the results of our more precise single-cell sequencing-based differential gene analysis. Additionally, we successfully captured the dynamic changes in cuproptosis features along the tumor progression trajectory for the first time. We discovered an increasing trend of resistance to cuproptosis along with an upward trend of CDKN2A expression. However, statistically, epigenetic modifications and mutations had minimal impact on its expression, except for an observed increase in chromatin accessibility. By utilizing PySCENIC, we identified MEF2D as a potential transcription factor involved in the regulation of CDKN2A transcription. Previous studies have reported that MEF2D overexpression promotes EMT and metastasis in colorectal cancer [45]. In gastric cancer, MEF2D activates the Wnt/β-catenin pathway to promote invasion [46]. Therefore, MEF2D may transcriptionally regulate CDKN2A, thereby facilitating disease progression. Additionally, increasing literature indicates that ceRNAs play a crucial regulatory role in biological processes. Some cuproptosis-related ceRNA regulatory axes have been discovered, such as XIST/miR-125a-5p/CDKN2A [47], MIR31HG/miR-193a-3p/TNFRSF21 [48], miR-432-5p/DLD [49]. In this study, we discovered the involvement of SNHG7/miR-133b in the regulation of CDKN2A, which was preliminarily validated through cellular experiments. These findings provide insights into the transcriptional and post-transcriptional regulatory mechanisms that influence CDKN2A overexpression, ultimately impacting cuproptosis and tumor progression.

Although cuproptosis depends on mitochondrial respiration and the TCA cycle, glycolysis can enhance its resistance [44, 50]. In a recent study conducted on melanosomes, the expression of CDKN2A was significantly downregulated upon inhibition of glycolysis [51]. Similar to our study, our research demonstrates that tumor cells expressing CDKN2A exhibit higher glycolytic levels. Silencing CDKN2A results in reduced expression of the rate-limiting enzyme phosphofructokinase-1, which is activated by fructose-2,6-bisphosphate. This suggests that CDKN2A suppresses cuproptosis by redirecting glycolytic flux. This metabolic deviation, which is distinct from iron-dependent cell death, promotes substrates that contribute to malignancy [39]. It is also associated with cuproptosis resistance and the progression of tumor malignancy.

The disruption of copper homeostasis is essential for triggering cuproptosis. Previous studies have speculated on a potential association between CDKN2A and copper homeostasis in cuproptosis. However, no supporting evidence was provided [52, 53]. However, a report indicates a positive correlation between the methylation levels of CDKN2A’s CpG islands and peripheral blood copper ion concentrations in occupations with copper exposure [54]. Studies have demonstrated an increase in CDKN2A levels in colorectal cancer cell lines following treatment with elesclomol-Cu [55]. We further elucidated the specific relationship between CDKN2A and copper by discovering that silencing CDKN2A leads to increased intracellular Cu^2+^ levels and alters the expression of copper transport genes SLC31A2 and ATP7B. ATP7B, a copper-transporting ATPase, facilitates the efflux of copper from the cell or its transport into the lysosomal lumen, resulting in the excretion of copper ions from the cell. Conversely, SLC31A2 transports copper ions from the lysosome back into the cytoplasm [56]. CDKN2A is able to prevent copper overload by regulating these transport proteins, thereby inhibiting cuproptosis. Noteworthy, lysosomes play a role in degrading toxic proteins and are involved in cellular stress mitigation through autophagy [57, 58]. However, whether this process can mitigate the toxic protein stress in cuproptosis remains unexplored, and research in this area is still lacking. Investigating whether CDKN2A mediates the crosstalk between autophagy and cuproptosis has also become the focus of our ongoing research.

CDKN2A is recognized as a tumor suppressor in certain cancers, such as breast cancer [59], where it exerts its influence by regulating the cell cycle to inhibit excessive proliferation of cancer cells. However, the effects of CDKN2A appear to vary depending on the tumor type. Notably, in colorectal cancer, CDKN2A has been found to promote disease progression by facilitating epithelial-mesenchymal transition [41]. In the present study, we proposed an alternative perspective on the tumor-promoting role of CDKN2A, suggesting that it may achieve this function by modulating intracellular copper ion concentration, potentially through the regulation of copper ion transport channel proteins. Furthermore, previous research has demonstrated that intracellular copper ions can exert anticancer effects through various pathways [60]. CDKN2A may act to counteract this inhibition. However, a comprehensive understanding of the underlying mechanisms necessitates further investigation.

The CDKN2A gene has been shown to play a pivotal role in regulating physiological levels of Wnt signal transduction [61, 62]. Previous studies have demonstrated that the bisulfiram/Cu complex can inhibit the Wnt/β-catenin signaling pathway in gastric cancer [63]. CDKN2A may mediate the observed activation of the Wnt pathway by inhibiting copper ion concentration. Its expression is also correlated with EMT, which is consistent with the previously reported link between CDKN2A and EMT [41, 64]. Furthermore, previous studies have reported that the EMT process can be influenced by the Wnt signaling pathway and MMP7 [42, 43]. It is noteworthy that EMT is often accompanied by metabolic reprogramming, leading to a preference for glycolysis in cellular metabolism [65, 66].

Studies have associated high CDKN2A expression with worse clinical outcomes in some tumors [9, 67] Similarly, we found that high CDKN2A expression correlates with poorer prognosis and clinicopathological stage. By analyzing colorectal cancer cell lines, we observed that 5-FU-sensitive cell lines have the characteristic of high CDKN2A expression during chemotherapy and radiotherapy. 5-FU inhibits growth by forming complexes with thymidylate synthase via its metabolite 5-fluorodeoxyuridine monophosphate, preventing DNA synthesis. The 5-FU metabolites FUTP and FUMP are also incorporated into RNA/DNA, causing mutations and breaks leading to cell death [68]. Similarly, radiation disrupts the DNA structure, resulting in unrepaired damage and apoptosis [69]. Therefore, this is likely related to the aforementioned upregulation of CDKN2A accompanied by active nucleic acid metabolism and high mutation rates of DNA damage repair genes. CDKN2A can be a potential biomarker for monitoring prognosis and chemotherapy/radiotherapy response. This provides a basis for using CDKN2A in colorectal cancer diagnosis, treatment, and prognosis.

A recent study has revealed the TME phenotype associated with cuproptosis-related molecular patterns [70]. In hepatocellular carcinoma, CDKN2A expression is associated with tumor purity and macrophage expression [9]. TAMs have been identified as regulators of metabolism and promoters of invasion. They secrete MMP7, a proteinase that degrades the extracellular matrix [71–73]. In a recently constructed single-cell sequencing-based colorectal cancer myeloid cell atlas, a subtype of TAMs derived from monocyte-like cells, known as SPP1^+^ TAMs, was discovered to be associated with a poorer prognosis [13, 27]. Notably, SPP1 serves as both a marker gene for TAM and a hallmark gene for tumor EMT [74]. pDCs exhibit a dual role in tumors, involving both immunosuppression and tumoricidal effects [75]. We further detected specific signaling differences between CDKN2A-expressing regions and adjacent regions, such as VEGF, PDGF, ANNEXIN and TWEAK. The VEGF and PDGF pathways can promote angiogenesis, supporting growth [76]. TWEAK signaling activates proliferation and invasion in prostate cancer, promoting mouse tumor growth and angiogenesis [77, 78]. These results suggest that CDKN2A expression is associated with immune infiltration, abnormal tumor-matrix signaling, and potential mediation of metabolic reprogramming by immune cells. The TME provides a new perspective for understanding the comprehensive mechanisms underlying how CDKN2A promotes tumor progression and resistance to cuproptosis.

Our study possesses several advantages. To the best of our knowledge, this is the first comprehensive study exploring the potential mechanisms of CDKN2A-mediated resistance to cuproptosis, supported by biological experiments. Unlike previous articles that were restricted to bioinformatics predictions [52]. We have conducted experimental validations to confirm the effects of CDKN2A on glycolysis and intracellular copper ions. Specifically, we have investigated CDKN2A’s impact on copper homeostasis, which has received limited prior research attention. Moreover, we have successfully elucidated the relationship between CDKN2A and the TME. In contrast to previous studies relying on bulk sequencing, our data are derived from spatial transcriptomics, which incorporates spatial information and offers a more precise and accurate depiction of cellular components and signaling interactions within the TME. Additionally, our findings have been corroborated by independent datasets, ensuring the reliability and feasibility of our results.

One of the limitations of our study is that, except for the single-cell queue, the sample data from other queues did not achieve single-cell resolution, which could introduce potential bias into the results. Moreover, our investigation of the mechanism underlying CDKN2A-mediated resistance to cuproptosis primarily focused on the factors triggering cuproptosis. Therefore, it is worthwhile to conduct further research on CDKN2A’s potential mechanisms to counteract and control cuproptosis when it occurs, such as its ability to counteract protein toxicity stress. Remarkably, this aspect remains unaddressed not only in our study but also in most studies related to cuproptosis. Real-time monitoring of cellular responses may be necessary during induced cuproptosis, and a recently developed sequencing technology called Live-seq could potentially provide valuable insights for future research in this area [79]. It is noteworthy that our study is constrained by its confinement to cell experiments and colorectal cancer. Future endeavors encompassing animal models and extending into other cancer types could yield more comprehensive evidence supporting our findings and potentially uncover novel insights. Our research posits that in forthcoming endeavors, the judicious combination of CDKN2A inhibitors with cuproptosis inducers may hold therapeutic potential for the treatment of colorectal cancer.

## CONCLUSIONS

In summary, our research elucidates the potential mechanism by which CDKN2A promotes resistance to cuproptosis and explores the relationship between CDKN2A and adverse prognosis. Furthermore, we provide valuable treatment strategies for cuproptosis-resistant colorectal cancer.

## Supplementary Material

Supplementary Figures

Supplementary Tables
