# Supplementary material for: The crosstalk role of CDKN2A between tumor progression and cuproptosis resistance in colorectal cancer
Source: Aging (Albany NY). 2024 Jun 17;16(12):10512–38. doi: 10.18632/aging.205945 (PMC11236303; doi:10.18632/aging.205945)
Supplement: Supplementary Figures [file aging-16-205945-s001.pdf]

SUPPLEMENTARY FIGURES

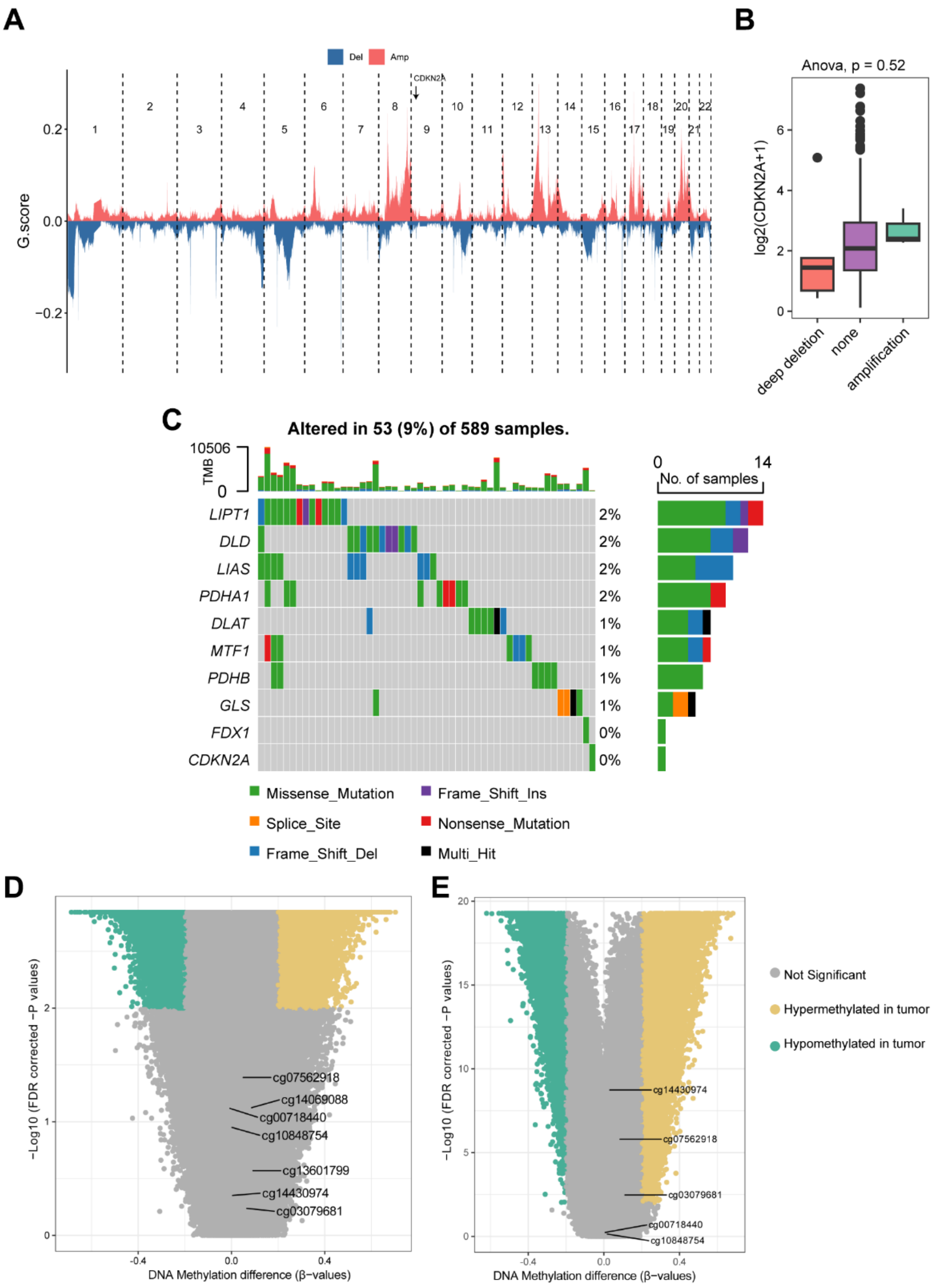

Supplementary Figure 1. Mutations and methylation status of cuproptosis-related genes in the TCGA cohort of CRC. (**A**) Copy number variation (CNV) G-score plots for all samples, with the Y-axis representing the G-score value and the X-axis representing the genomic position coordinates. The different colors indicate gene deletion or amplification events. (**B**) Comparison of CDKN2A expression levels among the samples with deep deletion, amplification, and no change. Statistical analysis was performed using ANOVA. (**C**) Single nucleotide polymorphism (SNP) mutation profile for all samples, depicting the presence of mutations in the ten cuproptosis-related genes. (**D**) Differential analysis of methylation sites between colorectal cancer and normal colon samples, with annotated probes targeting CDKN2A. (**E**) Differential analysis of methylation sites between rectal cancer and normal colon samples, with annotated probes targeting CDKN2A.

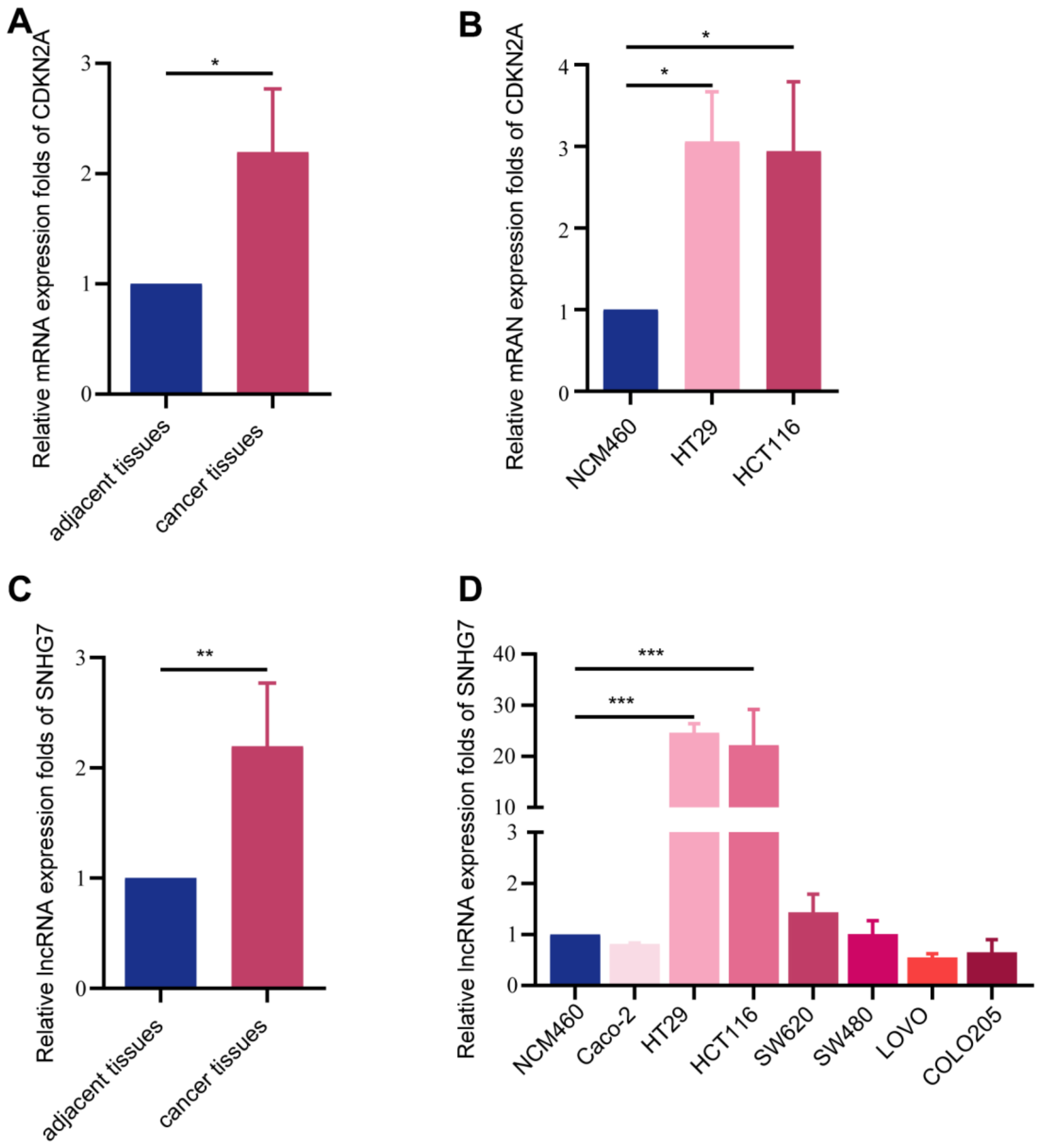

Supplementary Figure 2. Differential expression of CDKN2A and SNHG7 in colorectal cancer tissues and cell lines. (**A**) The differential expression of CDKN2A between 31 paired samples of colorectal cancer tissues and adjacent noncancerous tissues was determined using qRT–PCR analysis. (**B**) qRT–PCR analysis was performed to assess the differential expression of CDKN2A between the colon epithelial cell line NCM460 and different colorectal cancer cell lines. (**C**) qRT–PCR analysis was employed to determine the differential expression of SNHG7 in 31 paired samples of colorectal cancer tissues and adjacent noncancerous tissues. (**D**) The differential expression of SNHG7 between the colon epithelial cell line NCM460 and various colorectal cancer cell lines was determined using qRT–PCR analysis.
