# Supplementary material for: The crosstalk role of CDKN2A between tumor progression and cuproptosis resistance in colorectal cancer
Source: Aging (Albany NY). 2024 Jun 17;16(12):10512–38. doi: 10.18632/aging.205945 (PMC11236303; doi:10.18632/aging.205945)
Supplement: Supplementary Tables [file aging-16-205945-s002.pdf]

SUPPLEMENTARY TABLES

Supplementary Table 1. Primers used for quantitative RT - PCR analysis.

Genes		Primer sequences（5’ - 3’）
SNHG7	Forward	AACCTGTGAGGATCGGTCAG
SNHG7	Reverse	GTCACCTCCTTCCCGTGTTA
CDKN2A	Forward	GATCCAGGTGGGTAGAAGGTC
CDKN2A	Reverse	CCCCTGCAAACTTCGTCCT
WNT2B	Forward	CCTGTAGCCAGGGTGAACTG
WNT2B	Reverse	CGGGCATCCTTAAGCCTCTT
WNT3	Forward	ATGAACCGCCACAACAACGAGG
WNT3	Reverse	GTCCTTGAGGAAGTCACCGATG
WNT4	Forward	GGAACTGCTCCACACTCGACTC
WNT4	Reverse	CGCACATCCACAAACGACTGT
WNT5B	Forward	AAGGAGTTTGTGGATGCCC
WNT5B	Reverse	GCTACGTCTGCCATCTTATACAC
WNT9B	Forward	AGTGCCAGTTTCAGTTCCG
WNT9B	Reverse	GGAAAGCTGTCTCTTTGAAGC
WNT10	Forward	CCCAATGACATTCTGGACCT
WNT10	Reverse	TAAGCGGTGCAGCTTCCTAC
DLAT	Forward	GCAGGACTCATCACACCTATTGT
DLAT	Reverse	GTAGTTTACCCTCTCTTGCTTTGG
LIAS	Forward	GTATGTGAGGAAGCTCGATGTC
LIAS	Reverse	CACCCATCAACATGATCGTGG
SLC31A2	Forward	ATCAGCCAGCAGACCATCGCAG
SLC31A2	Reverse	TGAAGTAGCCGATGACCACCTG
SLC31A1	Forward	GGGGATGAGCTATATGGACTCC
SLC31A1	Reverse	TCACCAAACCGGAAAACAGTAG
FDX1	Forward	TTCAACCTGTCACCTCATCTTTG
FDX1	Reverse	TGCCAGATCGAGCATGTCATT
PFKL	Forward	AAGAAGTAGGCTGGCACGACGT
PFKL	Reverse	GCGGATGTTCTCCACAATGGAC
PFKM	Forward	AGCGTTTCGATGATGCTTCAG
PFKM	Reverse	GGAGTCGTCCTTCTCGTTCC
ATP7B	Forward	ATATTGAGCGGTTACAAAGCACT
ATP7B	Reverse	TGCCCCAAGGTCTCAGAATTA
GAPDH	Forward	CTTCTCCTTCAGGGCATCA
GAPDH	Reverse	CTTCTCCTTCAGGGCATCA

Supplementary Table 2. Transient transfection of gene sequences.

Name		Primer sequences (5` - 3`)
U6	sense	CTCGCTTCGGCAGCACA
	sense	AACGCTTCACGAATTTGCGT
SNHG7-siRNA	sense	GGCCUGACUACUUGCAATT
	antisense	UUGCAAGAAUGUCAGGCCTT
CDKN2A-siRNA	sense	GCCACGCACCGAAUAGUTT
	antisense	ACUAUUCGGUGCGUUGGGCTT
Has-miR-133b mimic	sense	UUUGGUCCCCUUCAACCAGCUA
	antisense	GCUGGUUGAAGGGGCAAAUU
Hsa-miR-133b inhibitor	sense	UAGCUGGUUGAAGGGGACCAAA
	nc	CAGUACUUUUGUGUAGUACAA
